# Genetic Diversity and Local Connectivity in the Mediterranean Red Gorgonian Coral after Mass Mortality Events

**DOI:** 10.1371/journal.pone.0150590

**Published:** 2016-03-16

**Authors:** Joanna Pilczynska, Silvia Cocito, Joana Boavida, Ester Serrão, Henrique Queiroga

**Affiliations:** 1 Departamento de Biologia and CESAM - Centro de Estudos do Ambiente e do Mar, Universidade de Aveiro, Aveiro, Portugal; 2 Department of Earth and Environmental Sciences, University of Pavia, Pavia, Italy; 3 ENEA, Marine Environment Research Centre, La Spezia, Italy; 4 CCMAR - Centro de Ciências do Mar, Universidade do Algarve, Faro, Portugal; University of Padova, ITALY

## Abstract

Estimating the patterns of connectivity in marine taxa with planktonic dispersive stages is a challenging but crucial task because of its conservation implications. The red gorgonian *Paramuricea clavata* is a habitat forming species, characterized by short larval dispersal and high reproductive output, but low recruitment. In the recent past, the species was impacted by mass mortality events caused by increased water temperatures in summer. In the present study, we used 9 microsatellites to investigate the genetic structure and connectivity in the highly threatened populations from the Ligurian Sea (NW Mediterranean). No evidence for a recent bottleneck neither decreased genetic diversity in sites impacted by mass mortality events were found. Significant IBD pattern and high global F_ST_ confirmed low larval dispersal capability in the red gorgonian. The maximum dispersal distance was estimated at 20–60 km. Larval exchange between sites separated by hundreds of meters and between different depths was detected at each site, supporting the hypothesis that deeper subpopulations unaffected by surface warming peaks may provide larvae for shallower ones, enabling recovery after climatically induced mortality events.

## Introduction

Extreme weather events, including floods, heat waves and droughts, are currently emerging as one of the most important facets of climate change, and a growing body of literature is focused on extreme events [1]. Anomalous and extreme events due to global warming have increased considerably during recent decades in temperate regions such as the Mediterranean Sea and an increase in the frequency of heat wave extremes of 200–500% is predicted at the end of the twenty-first century [2]. Extreme events, together with other sources of mortality caused by human impact, such as overfishing and environmental pollution, may cause significant impacts on genetic diversity as a result of population size decrease [3], as has been observed after mass mortality events [4; 5].

If natural populations consist of reduced numbers of individuals, loss of genetic variability may dramatically influence the populations themselves, since genetically depauperate populations might fail to adapt to future environmental changes, eventually causing their disappearance. Due to the predicted intensification of weather extremes, many species may not have sufficient genetic potential for the evolution of strategies able to mitigate their impact. Nevertheless, genetic diversity reduction after mass mortality events is not a rule in the marine realm [3; 6]. There is an urgent need to carry out dedicated research to acquire more extensive, sound data across a range of life-history and demographic features, with the ultimate aim of formulating better predictions about the role of catastrophic disturbances in determining genetic structure and genetic diversity.

Connectivity patterns and gene exchange among populations are major research topics in marine ecology and are essential for the planning of marine reserves [7], to function as interconnected networks that can supply recruits to sites that undergo population bottlenecks or local extinctions. Genetic recovery from events of mass mortality, either natural or human-induced, may be dependent on the possibility of dispersal from external sources or from small local populations at refugial pockets [8–10]. This can result in much slower recovery of genetic diversity than population density, because demographic recovery is possible from a few founder or bottleneck survivors but genetic recovery requires extensive levels of connectivity or a long period of time [4]. However, inferring levels of connectivity is particularly challenging in the marine environment, where many species disperse exclusively by means of planktonic propagules, such as larval or spore stages, in a 3-dimensional fluid medium. Direct estimation of dispersal by tracking marine propagules is often not feasible in most instances, because of their small size and the unbounded nature of the marine environment [11; 12], so indirect methods, such as the use of neutral genetic markers [13; 14], are commonly applied.

The red gorgonian *Paramuricea clavata* (Risso, 1826) is a key species of sublittoral rocky habitats [15], widespread in the western Mediterranean Sea and in the Adriatic Sea [16] and less common in the Aegean Sea [17]. The species is a surface brooder with a short larval dispersal phase [18; 19]. In situ observations indicate that the dispersive stage of the larvae may last only a few minutes and thus the larvae are expected to settle near the mother colony [18]. This mechanism does not favor dispersal, but possibly decreases larval mortality and wastage, contributing to the replenishment of local populations [20]. However, metamorphosis may be delayed in the laboratory for up to 25 days after egg collection, suggesting high dispersal capacity, at least under certain conditions [18]. In 1999 and 2003, two episodes of mass mortality, connected with increased water temperature reaching 24°C [21], affected several populations of benthic suspension feeders in the northwestern Mediterranean, causing a drastic decrease of *P*. *clavata* colony density [22–25]. The number of colonies affected by partial mortality decreased with depth [22; 23; 25], because mortality affected the community from the surface to approximately 20 m, the approximate depth of the thermocline. Hereafter we designate as deep the sites with colonies living below the thermocline, which were not exposed to abnormally long lasting summer conditions [26]. Therefore, the clear differences in the number of damaged colonies and the extent of injury were visible between shallow populations (dwelling above the thermocline) and the deep ones, even when the depth difference was only of a few meters [22; 25]. As hypothesized by Cerrano & Bavestrello [27; 28], the deep dwelling subpopulations may act as a reservoir, supplying larvae to shallower sites, especially because large colonies with high fecundity rates survived there. However, little is known about short distance dispersal of the species. Surveys based on microsatellite loci in the Mediterranean Sea, showed that *P*. *clavata* exhibits a high level of genetic differentiation at the small and large spatial scales, which is consistent with the short larval dispersal displayed by the species [29–31]. Connectivity among populations of *P*. *clavata* separated by less than 14 km was weak in the study of [31], since the mean immigration rate was below 9% and most of the immigrants came from neighboring populations located only hundreds of meters away.

In the present study we used microsatellite markers to study the genetic structure of *P*. *clavata* populations in an area impacted by mass mortality events, in the Ligurian Sea, Mediterranean. Our objectives were twofold. The first objective was to investigate a possible bottleneck effect of past mortality events on genetic diversity in a region where some populations were highly affected by mass mortality events and others were not. In this case we are expecting to detect the decrease of genetic diversity at sites impacted by mortality. Our second objective was to understand the connectivity patterns and migration at the scale of a few tens of km since up to date very few studies have examined short distance migration in the species. We expect to detect migrations between non-impacted reefs and impacted ones, supporting recovery of damaged populations. Our research contributes to better understand the mechanisms that enable recovery of threatened populations by providing data about larval migrations between impacted and healthy populations of the red gorgonian. The results should prove to be particularly valuable for the conservation of soft corals communities and thus the overall marine biodiversity.

## Material and Methods

### Sampling

**Fig 1:**
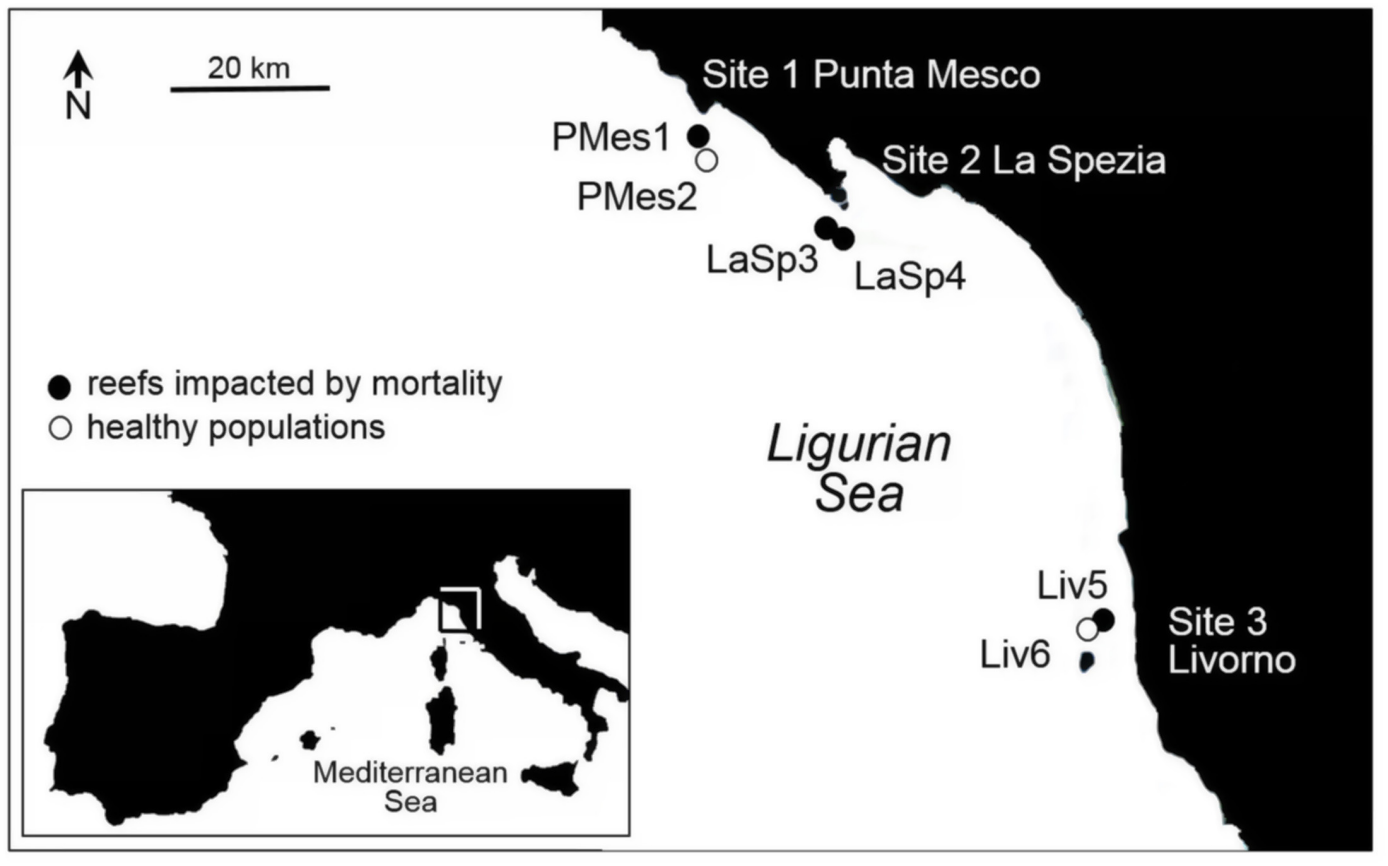
Sampling sites in the Mediterranean. Reefs impacted by mass mortality events (in black) and healthy reefs (in white).

*P*. *clavata* colonies were sampled by scuba divers following a hierarchical sampling design. Samples were taken from three sites, Punta Mesco, La Spezia and Livorno, in the Ligurian Sea (NW Mediterranean). At each site two different reefs were chosen (Fig 1, Table 1). In the remaining of the text, each reef will be referred to by its code from Table 1. At Punta Mesco and Livorno reefs were sampled at different depths. At these two sites shallow reefs (<25 m depth) were impacted by mass mortality events, whereas colonies dwelling below 25 m (referred as deep reefs in the remaining of the text) remained non-impacted ([32]; Di Fiore M., pers com). At La Spezia both reefs were sampled at the same depth, since the rocky cliff ends on a muddy bottom at 24–25 m, and therefore both reefs were damaged during the mass mortality (Table 1). Populations impacted by mass mortality recovered significantly. Colonies density in La Spezia population declined from over 35 to nearly 8 colonies m^-2^ shortly after the mass mortality and increased to 20 colonies m^-2^ four years later [33]. In Portofino (Ligurian sea), located near Punta Mesco, colonies density decreased after the mortality event from nearly 20 to 5 colonies m^-2^, but 3 years after the event the density recovered to pre-mortality levels [23]. We do not have data from Punta Mesco and Livorno but we may expect that the recovery pattern was similar and, therefore, all impacted populations consist of survivors and new colonies that settled after the mortality. Sites were separated by distances ranging from 20 to 60 km (Fig 1) whereas reefs within each site were separated by 200 to 300 m. Site 1, Punta Mesco, is located in the Cinque Terre Marine Protected Area. A small portion of colony branch (around 3–4 cm of branch tip) was taken from up to 30 different colonies randomly chosen from each reef. The branch tip from each colony was stored individually in a plastic tube underwater. Samples were placed on ice during transport and preserved in ethanol after arrival to the laboratory, no later than 3 hours after collection.

**Table 1:** Sampling sites characteristics.

*Site*	*Reef code*	*Depth [m]*	*Past mass mortality events*	*N*	Reef coordinates
1 Punta Mesco	PMes1	21–23	1999 and 2003 [32]	30	44°7’59”N 9°38’7”E
	PMes2	28–29	No [32]	30	44°7’59”N 9°38’9”E
2 La Spezia	LaSp3	18–22	1999 and 2003 [24; 34]	29	44°1’25”N 9°51’2”E
	LaSp4	19–20	1999 and 2003 [24; 34]	30	44°1’22”N 9°51’4”E
3 Livorno	Liv5	23–25	2006 (Di Fiore M, pers com)	25	43°27’50”N 10°19’48”E
	Liv6	30–31	No (Di Fiore M, pers com)	30	43°28’5”N 10°19’49”E

The depth range of sampled reefs, the year of past mass mortality events, N—the number of colonies sampled at each reef and the geographic coordinates.

The Cinque Terre Marine Protected Area authorized ENEA to conduct fieldwork and sampling at Punta Mesco (reef 1 and 2). La Spezia Islands (reef 3 and 4) are included in a recently established marine conservation area where ENEA is authorized by the Regional Natural Park of Porto Venere to conduct fieldwork and sampling. No specific permission was required for sampling at Livorno. *P*. *clavata* is not endangered nor protected.

### Microsatellite analysis

Coral DNA was extracted using E.Z.N.A. Mollusc DNA Kit according to the manufacturer handbook. We analyzed 10 microsatellite, developed by Agell *et al*. [35] and Mokhtar-Jamaï *et al*. [36] following the protocols published by the authors. Loci Par_a, Par_b, Par_d, Par_f and Par_m were amplified from total genomic DNA in 10 μl solution of dNTPs (0.25 mM each), selected primers (0.25 μM each), 4 mM of MgCl_2_, 1x manufacturer-supplied buffer and 0.25 u DFS-Taq DNA Polymerase (Bioron). The PCR program was: 2 min 94°C, (10 sec 94°C, 20 sec AT, 1 min 72°C)x30, 5 min 72°C. Annealing temperatures (AT): Par_a: 59°C, Par_b: 47°C, Par_d: 51°C, Par_f, Par_m: 52°C. To amplify loci Parcla_9, Parcla_10, Parcla_12, Parcla_14 and Parcla_17, total genomic DNA was dissolved in 10 μl solution of dNTPs (125 μM each), selected primers (0.5 μM each), 0.25 u GoTaq^®^ DNA Polymerase (Promega) and 1x manufacturer-supplied PCR buffer. The PCR program was: 3 min 94°C, (1 min 94°C, 1 min 60°C, 1 min 72°C)x30, 5 min 72°C. The length of amplified fragments was analyzed on an ABI 3730XL Genetic Analyzer using an internal size standard (GeneScan 500 LIZ). The analysis of DNA fragment length was performed with STRand [37]. Scored microsatellite fragment sizes were then visualized in R environment using the MsatAllele_1.02 package to track and reanalyze scoring errors.

### Genetic diversity

MICRO-CHECKER v.2.2.3 [38] was used to estimate null allele frequency and to check for scoring errors owing to stutters and large allele dropout. Linkage disequilibrium among all pairs of loci was tested in GENEPOP 4.2. [39; 40] with significance levels determined by the Markov chain method (dememorization = 5000, batches = 500, iterations = 10 000).

Observed (H_o_) and Nei’s [41] unbiased expected heterozygosity (H_e_) were computed in GENETIX v.4.05 [42]. The rarefaction procedure implemented in HP-RARE software [43] was used to estimate allelic richness (A_r_) and private allelic richness (A_p_). The minimum number of genes was set to 25 (the minimum sample size). Differences in heterozygosity and allelic richness between impacted and healthy reefs were tested using Kruskal-Wallis. The power of the test and minimum sample size to achieve 90% power were calculated in PASS 14 [44]. Single and multi-locus Weir & Cockerham’s [45] *f* estimator of F_IS_ were calculated using GENEPOP 4.2. Departures from Hardy–Weinberg (HW) equilibrium within sample for each locus and over all loci were tested in GENEPOP 4.2. The level of significance was determined by the Markov chain method using the default parameters (dememorization = 1000, batches = 100, iterations = 1000).

Populations that experienced a recent mass mortality are predicted to lose allelic diversity faster than heterozygosity [46] and thus exhibit a heterozygosity excess relative to the heterozygosity expected from the observed number of alleles. To establish whether there is a heterozygosity excess or deficit, the BOTTLENECK software [47] computes a distribution of the expected heterozygosity under the assumption of mutation-drift equilibrium, calculated from the observed number of alleles, and compares it to the heterozygosity expected under Hardy-Weinberg equilibrium. The presence of a possible bottleneck effect was tested using 9999 simulations under the Two-Phase Model (TPM), since it generally fits microsatellite evolution better than either pure stepwise or infinite allele models [46, 47]. TPM assumes that the majority of mutations are single steps, when alleles increase or decrease by one repeat unit. The mutation sizes for remaining mutations are drawn from a geometric distribution and larger mutations are rare, but do occur. The frequency of step mutations was set to 0.9 (ps), the variance of mutations to 12 and the Wilcoxon test was used to test the null hypothesis of no significant heterozygosity excess.

### Population differentiation and connectivity

Global and pairwise Weir & Cockerham’s [45] estimator of F_ST_ was estimated in GENEPOP. The genotypic differentiation between all pairs of reefs was tested in GENEPOP with default parameters.

The isolation by distance pattern was tested in GENEPOP. The shortest possible distance over sea between each reef pair was measured using Google Earth 7.1.2.2041. The relationship between genetic distance [F_ST_/(1-F_ST_)] and spatial distance [km] was tested using a Mantel test (n = 2000).

In order to quantify genetic variation within reefs, among reefs within a site and among sites a hierarchical analysis of molecular variance (AMOVA) was performed in ARLEQUIN 3.5 [48]. The significance of these variance components was tested using 50000 permutations.

The Bayesian approach implemented in STRUCTURE v.2.2 was used to investigate population structure. The recessive allele option was used to deal with null alleles [49]. The number of clusters (K) in the data set was evaluated under the admixture model with correlated allele frequencies. First run of 10 iterations, burnin of 10000 and MCMC = 50000 was computed for K from 1 to 10. The value of K that captures the major structure in the data was selected based on the plot of logarithm of the likelihood of observing the data [LnP(D)] as a function of K [50]. STRUCTURE was then run 30 times for K values from 2 to 6. The results were merged in CLUMPP [51] and graphically displayed in DISTRUCT [52]. The analysis was then repeated in the groups defined in the first run of STRUCTURE, when K = 3, to search for substructure within the groups.

A Bayesian assignment method [53] implemented in GENECLASS2 [54] was used to detect putative first generation migrants (F0). A Monte Carlo resampling method, as described in Paetkau *et al*. [55], was performed to evaluate each individual’s probability of belonging to a population from each reef.

Whenever multiple tests were conducted (linkage disequilibrium, HW equilibrium, genetic differentiation), the level of significance was adjusted using a false discovery rate (FDR) [56].

## Results

### Genetic diversity

No large allele dropout was detected by MICRO-CHECKER at any locus, but evidence of scoring errors due to stuttering was found in Parcla_12 and this locus was excluded from further analysis. The mean null allele frequency across all reefs varied from 0 for Parcla_10, Parcla_17 and Par_f to 0.18 for Par_m. No significant linkage disequilibrium was observed between any pair of loci (all p>0.05 after FDR correction), thus all loci were considered as genetically independent.

Eight of the loci were polymorphic at all sites, whereas one locus, Par_b, was monomorphic at LaSp3 and LaSp4 according to the 0.95 frequency criterium. The total number of alleles ranged from 4 for Par_b, Par_d and Par_f to 27 for Par_m. Unbiased expected heterozygosity (H_e_) varied between 0.57 at PMes1 to 0.66 at Liv6, with a mean value of 0.62 (0.03). H_o_ ranged from 0.42 for LaSp4 to 0.59 at Liv5, with a mean value of 0.51 (0.07) (Table 2). The lowest allelic richness (A_r_) was found at PMes1 (4.77) whereas the highest value was found at PMes2 (5.84). Private allelic richness (A_p_) varied from 0.34 at PMes1 to 1.17 at PMes2 (Table 2). There was no evidence that reefs affected by mass mortality events had lower genetic diversity than healthy ones (Kruskal-Wallis, all p>0.05). The power of the test was low for He (20%) and Ar (20%), and moderate for Ho (70%) and Ap (66%). In our case, to achieve test power reaching 90% for the 0.05 confidence level, sample size would need to be at least 166 colonies per reef. Multilocus F_IS_ ranged from 0.08 at Liv5 to 0.31 at LaSp4 (Table 2). When all loci were examined separately, the lowest value was -0.26, for Par_b, and the highest one, 0.70, for Par_a.

**Table 2:** Measures of genetic diversity.

	He (± SD)	Ho (± SD)	Ar	Ap	F_IS_
**PMes1**	0.57 (± 0.28)	0.46 (± 0.27)	4.77	0.34	**0.20**
**PMes2**	0.61 (± 0.27)	0.48 (± 0.23)	5.84	1.17	**0.20**
**LaSp3**	0.60 (± 0.25)	0.52 (± 0.27)	4.92	0.55	**0.13**
**LaSp4**	0.61 (± 0.25)	0.42 (± 0.27)	4.93	0.39	**0.31**
**Liv5**	0.64 (± 0.18)	0.59 (± 0.23)	5.40	0.57	**0.08**
**Liv6**	0.66 (± 0.14)	0.57 (± 0.15)	5.18	0.47	**0.13**
*mean*	*0*.*62 (± 0*.*03)*	*0*.*51 (± 0*.*07)*	*5*.*17 (± 0*.*40)*	*0*.*58 (± 0*.*30)*	*0*.*17 (± 0*.*08)*

Measures of genetic diversity (mean ± SD) in 6 reefs of *Paramuricea clavata* at 9 microsatellite loci. H_e_−Nei’s [41] unbiased expected heterozygosity; H_o_—observed heterozygosity; Ar and Ap—allelic and private allelic richness, respectively (with rarefaction size of 14 genes); F_IS_—Weir & Cockerham’s [45] *f* estimator of F_IS_ with significant values in bold (0.05 threshold after FDR correction).

Significant heterozygote deficiency was detected at all reefs (Table 2). However, the departures from HW equilibrium were not evident for all loci at all sites. Populations from all reefs revealed a departure from HW equilibrium at locus Par_m, but the highest frequency of null alleles was found in this locus and evidence for null alleles was also detected at all reefs for this locus. When Par_m was excluded from the analyses, heterozygote deficiency was still significant at all reefs except Liv5 after FDR correction.

No evidence for a recent genetic bottleneck was detected for any of the investigated reefs. A heterozygote excess (an indicator of recent bottleneck) was not found at any reef (Wilcoxon, all p>0.58) neither was a heterozygote deficit (i.e., a sign of expansion) (all p>0.08).

### Population differentiation and connectivity

Global F_ST_ value was 0.118, whereas pairwise comparisons between all pair of reefs varied from -0.003 between Liv5 and Liv6 to 0.189 between PMes1 and Liv6 (Table 3). All comparisons were significant (p<0.05), except Liv5 and Liv6, which revealed also no statistical differences in genotypic composition (Chi2 = 18.5, df = 18, p = 0.42).

**Table 3:** Pairwise F_ST_ values.

	PMes2	LaSp3	LaSp4	Liv5	Liv6
**PMes1**	0.00411	0.08499	0.07483	0.18168	0.18917
**PMes2**		0.08048	0.06603	0.17626	0.18165
**LaSp3**			0.00483	0.17214	0.17452
**LaSp4**				0.16189	0.16454
**Liv5**					-0.00332

Global and pairwise Weir & Cockerham’s [45] estimator of F_ST_ between all pairs of *P*. *clavata* reefs. All pairwise comparisons were significant (p<0.05) except one (Liv 5 and Liv6).

**Fig 2:**
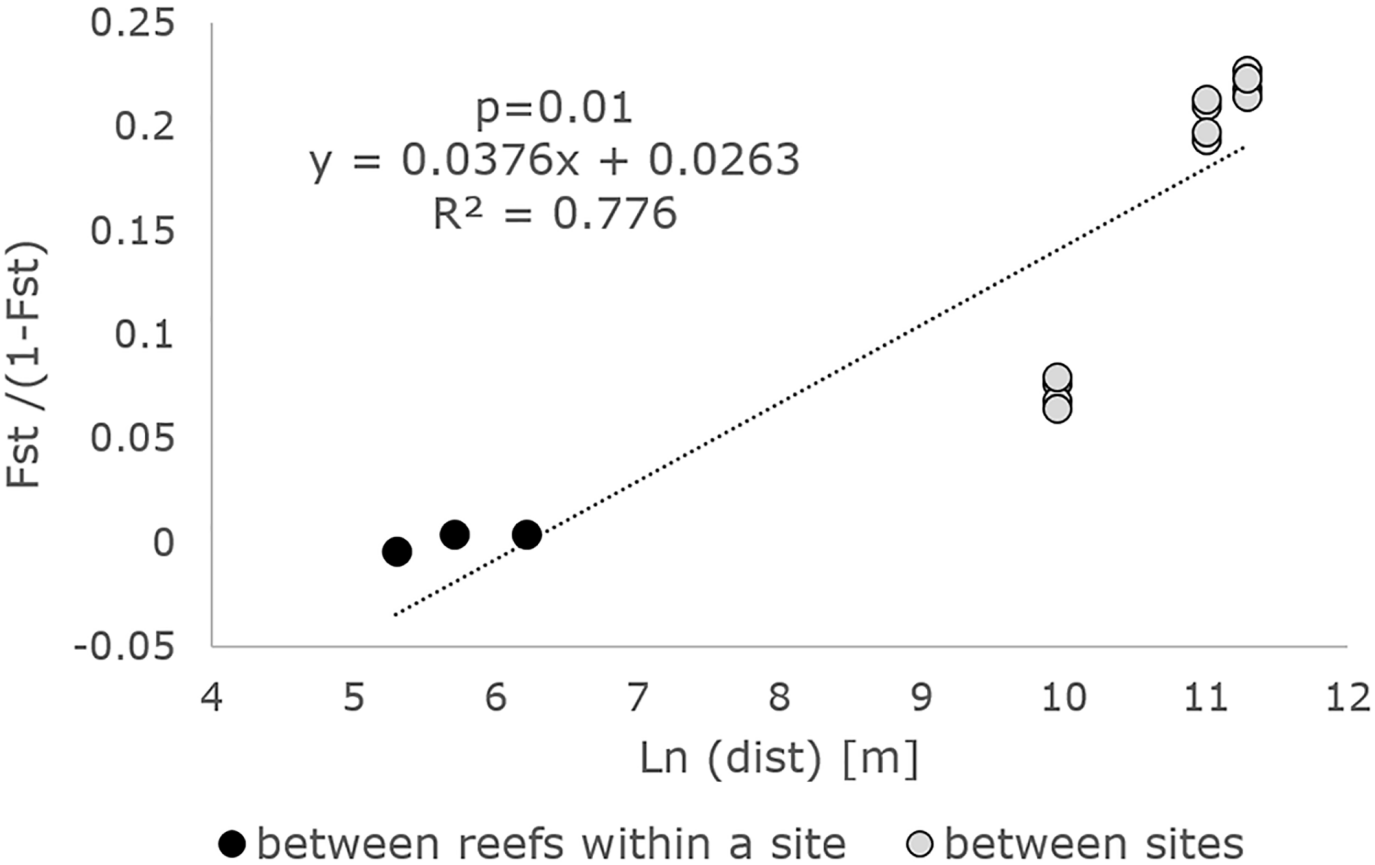
The isolation by distance pattern for *P*. *clavata*. Linear regression of the genetic distance measured as F_ST_ / (1—F_ST_) over the geographic distance (m).

The correlation between F_ST_/(1-F_ST_) and distance was significant (p = 0.01), supporting an isolation by distance model of gene flow in *P*. *clavata* at the local scale (Fig 2).

The AMOVA (Table 4) revealed that a highly significant percentage (11.19%) of the total genetic variation occurred among sites, whereas a smaller, but still significant, percentage of variation was explained by differences among reefs within sites (1.17%). Indeed, most of the variance was explained by differences within reefs (87.64%) and this was highly significant.

**Table 4:** AMOVA.

Source of variation	df	Sum of squares	Variance components	% of variance	*p*-value
Among sites	2	101.88	0.39229 Va	11.19	<0.0001
Among reefs within sites	3	16.36	0.04114 Vb	1.17	<0.05
Within reefs	342	1050.82	3.07257 Vc	87.64	<0.0001
Total	347	1169.05	3.506		

Analysis of molecular variance (AMOVA) among *P*. *clavata* reefs.

**Fig 3:**
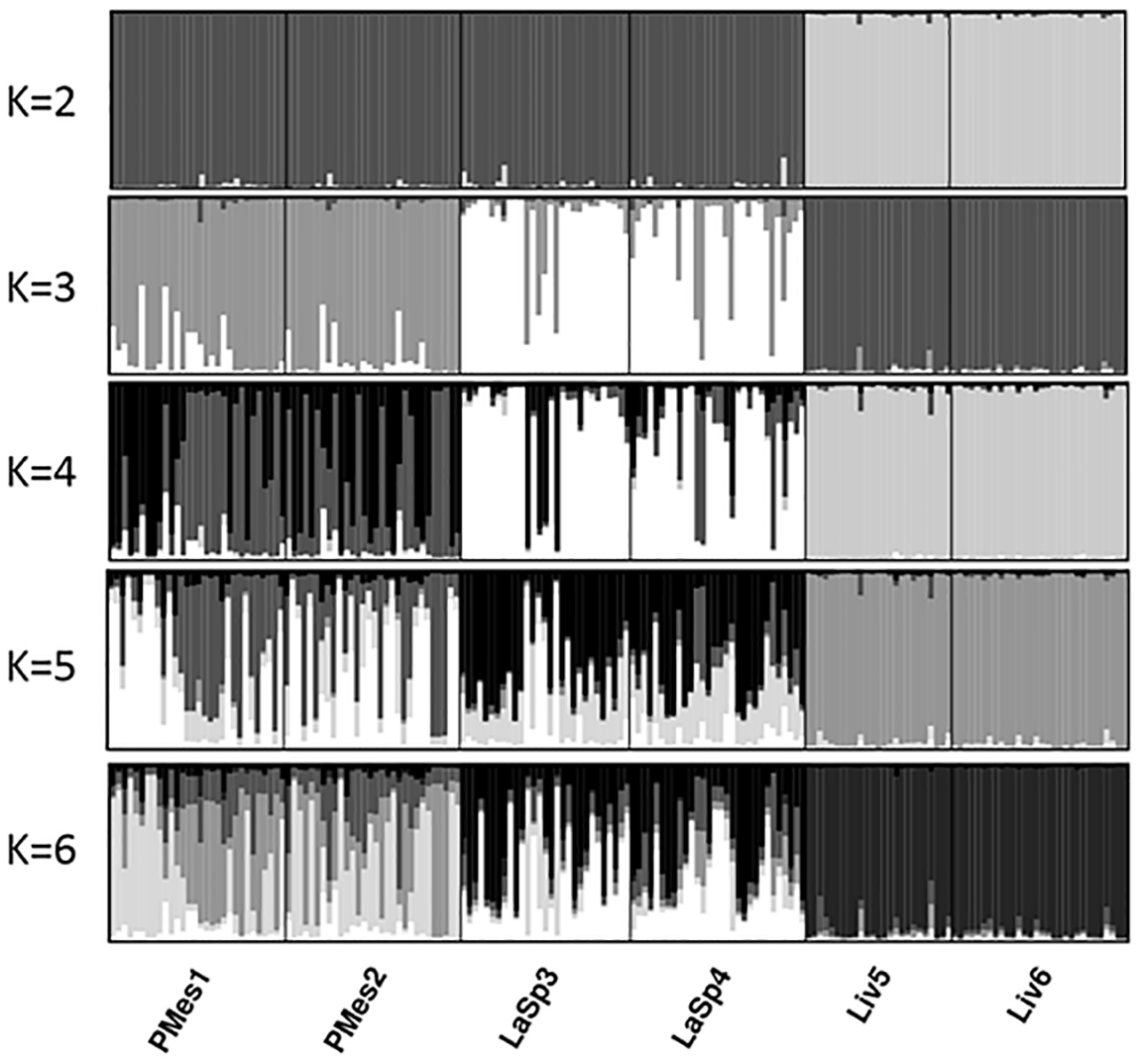
Clustering analysis. Population structure revealed by the first run of clustering analyses in STRUCTURE. Each individual is represented by a vertical line, divided into segments representing the proportion of the genome of the individual that is assigned to each cluster. The number of clusters was set to 2–6. Reefs are separated by a black vertical line.

In the STRUCTURE analysis the plot of LnP(D) as a function of K revealed a plateau for K≥3 (S2 Fig). Samples were divided into 3 clearly separated clusters, each of them grouping the two reefs from the same site (Fig 3). Samples from Livorno (Liv5 and Liv6) displayed a high coefficient of population membership while reefs from Punta Mesco (PMes1 and PMes2) and La Spezia (LaSp3 and LaSp4) showed a low level of admixture. A second run of STRUCTURE did not reveal any genetic structure within the groups (data not shown). When K was set to 2, reefs from Livorno were separated from the others, whereas a K value over 3 did not reveal any additional structure.

According to GENECLASS2, 37.9% of the colonies were first generation migrants (F0). Most of them (31.6%) were exchanged between reefs separated by 200–300 m within the same site. The contribution of F0 migrants from the same site varied from 23.3 to 31.0% for PMes1 and LaSp3, respectively (Table 5). Between-sites migration was detected only between La Spezia and Punta Mesco, separated by 20km. The data suggest that migration occurred in both senses, with 10.0% of the colonies from PMes (percentage from both reefs pooled together) assumed as migrants from LaSp and 8.5% of the colonies form LaSp contributed from PMes. Migrants exchanged between Liv5 and Liv6 were not evaluated because these sites do not differ significantly (F_ST_ = -0.00332).

**Table 5:** First generation migrants.

Source of migrants						
	PMes1	PMes2	LaSp3	LaSp4	Liv5	Liv6
PMes1	-	23.3	3.3	6.7	0.0	0.0
PMes2	30.0	-	3.3	6.7	0.0	0.0
LaSp3	3.4	0.0	-	31.0	0.0	0.0
LaSp4	3.3	10.0	26.7	-	0.0	0.0
Liv5	0.0	0.0	0.0	0.0	-	-
Liv6	0.0	0.0	0.0	0.0	-	-

The percentage of *P*. *clavata* colonies assumed as first generation migrants (F0) from each of the investigated reefs.

## Discussion

### Genetic diversity

Our results did not support the hypothesis of genetic effects of past mass mortality events in *P*. *clavata* from the Ligurian Sea. No differences in genetic diversity between reefs affected by mass mortality events and healthy ones were detected. Additionally, the levels of genetic diversity found in *P*. *clavata* from the Ligurian Sea were not much lower than the range of values reported previously for the species [29] and other Mediterranean [57] and tropical corals [58–60]. Numerous gorgonian populations in the Mediterranean were affected by mass mortality in the recent past [22–25] and there is the concern that these may have gone through genetic bottlenecks as a result of decreased population densities. However, confounding effects may obscure bottleneck results. For example, in *Corallium rubrum* (Linnaeus, 1758), 16 of 40 Mediterranean shallow populations showed a sign of recent expansion after a bottleneck [61], although a Wahlund effect could not be discarded. In a study of *P*. *clavata* from Ibiza (Balearic Islands, Spain), partial mortality, measured as the proportion of colony tissue damaged, was negatively correlated with effective population size, mean number of alleles per population and proportion of recent migration rates [31]. These results indicated that populations with colonies that are partially affected by mortality are less diverse, undergo a larger effect of drift and receive less immigrants than healthy gorgonian populations. Additionally, in our study, allelic richness was slightly higher in populations non-impacted by mass mortality, which is in agreement with published results [31]. Yet, in spite of the extensive density reductions that mass mortality events had on the Ligurian red gorgonian (ca. four-fold; see methods section), our results show no difference in genetic diversity between healthy and impacted sites. This may suggest that the adaptive potential of surviving populations was not reduced. Nonetheless, this interpretation should be cautious given that our sampling design was limited to six reefs, among which only two were not impacted by mass mortality. Consequently the test presents low to moderate power (20–70%) to detect differences between genetic diversity of healthy and impacted sites (high probability of making a type II error, i.e., failing to detect existing differences). The need to increase power in such tests with higher number of samples and loci has been highlighted in previous studies [62]. To achieve a power of 90%, we would need to sample 166 colonies per reef (ca. 1000 colonies in total), which is unrealistic, because of the logistic effort and the limited number of colonies at some reefs. It is also noteworthy that heterozygosity excess tests have been shown to have a limited power to detect mild-bottlenecks of 10-1000-fold population declines [63]. Even if past mass mortality events have affected genetic diversity, it may not be detected by our study, because of the lack of power of the statistical tests and other confounding factors such as recent expansion or Wahlund effect.

The departures from Hardy-Weinberg equilibrium found in the present research, indicated by high and significant F_IS_ values, are typical for species exhibiting low larval dispersal, and were already reported in *P*. *clavata* populations [29]. Heterozygote deficit as a result of inbreeding was previously reported in coral species characterized by having a short larval dispersal [58; 60; 64]. Our findings confirm previous studies and are in accordance with the reproductive biology and larval ecology of *P*. *clavata*. Planulae exhibit negative phototaxis and negative buoyancy and settle near the mother colony [18], increasing the probability of subsequent mating with closely related individuals, associated with reduced dispersal of the male gametes also. High F_IS_ values may also result from a high number of F0 migrants, analogously to Wahlund effect, since migrants come from genetically distinct population. The presence of null alleles may also partially explain the result, but heterozygote deficit was still prevalent in the absence of the main locus suspected to have null alleles.

### Genetic structure and connectivity

The high value of global F_ST_ indicates strong genetic differentiation among reefs and additionally supports the low dispersal capability of the larvae. The value obtained in the present study was nearly equal to the one previously found in the species (0.116 versus 0.118 in the present study) [29]. Our results indicated that the majority of variance in the population can be explained by the within reefs variation, indicating large variability among individuals. Although the variation among reefs within sites is smaller, reefs differ significantly in relation to the variability present at their respective site. Finally, variation among sites is high and significant as well. Therefore, the high diversity within each population should not be interpreted to conclude that populations differ only slightly. Higher differences between sites than between reefs within sites are consistent with a significant IBD pattern. Distance clearly acts as a barrier to gene flow in *P*. *clavata*, which is typical for species with short larval dispersal [33]. Moreover, isolation by distance is a phenomenon that occurs at local scales [65] and therefore it is evident among the closely located populations studied here. At larger spatial scales (hundreds to thousands of km), *P*. *clavata* also displayed a significant IBD pattern, similarly to another Mediterranean coral, *C*. *rubrum* [66]. However, at a fine spatial scale (cm to m), red gorgonian colonies did not show significant IBD, in contrast to *C*. *rubrum* [66].

Short distance migration (hundreds of meters) is likely to be the dominant scale of dispersal in *P*. *clavata*, but our results also suggest that migration from close undisturbed sites may be a significant source of recruits for disturbed areas to recover, since the maximum larval dispersal was between 20 to 60 km, with migrants detected in reefs separated by 20 km, but not by 60 km. This strengthens evidence from studies showing that coral population recovery after catastrophic mortality may be mainly supported by local migration from undamaged sites. A study of genetic connectivity in coral populations recovering after catastrophic bleaching revealed that the majority of detected migrants originated from the only site that was not decimated by a recent mortality event. Most of these immigrants were received by the site which reached pre-bleaching diversity, highlighting their role in population recovery [8]. Our results demonstrate that larvae may disperse from the Cinque Terre Marine Protected Area (Punta Mesco) to the adjacent populations, supporting their recovery after the disturbance. Additionally, the larval transport from deep, healthy reefs to the shallow ones, impacted by past mass mortality events, may have great importance for recovery after climatically induced population collapses.

Our findings indicate higher larval dispersal potential than reported in [19], which observed that larvae settle immediately on the substrate surrounding their mother colony. We found that transport of the larvae was not only common over distances of hundreds of meters, but also for tens of kilometers. The maximum migration distance detected in the present study reached 20 km, i.e. the distance that separates the two sites of La Spezia and Punta Mesco. Ten percent of the colonies from Punta Mesco were estimated as migrants from La Spezia. In this case, the larval dispersal was consistent with predominant currents, with the large scale Ligurian circulation being characterized by a cyclonic, east-to-west flow, active all year round and modulated by seasonality and wind forcing [67]. High contribution of migrants from Punta Mesco (8.5%) found in La Spezia may be caused by the predominantly southwesterly summer wind (Libeccio), which occasionally reverses coastal current direction [68]. In contrast to the previous two sites, Livorno was genetically homogeneous. This site is separated from La Spezia by 60 km of sandy bottom, unsuitable for the red gorgonian. The nearest known population to the south is located around 25 km from Livorno, at the deep rocky shoal below 40–50 m. It seems that both distance and depth differences may isolate Livorno reefs from other populations.

Theoretically, the high number of migrants exchanged between Punta Mesco and La Spezia should lead to panmixia in a few generations [69], but the genetic structure between these sites remains relatively high. These observations may be explained by an increased recruitment rate after the mortality. In stable populations the number of recruits is low, but after mass mortality the amount of available substrate increased and was occupied by new settlers, including migrants. When colonies recruited after the mass mortality events reached maturity and started reproducing, most of the available substrate was already occupied and only a minor proportion of their offspring could settle and survive, effectively reducing the opportunity for successful out-crossing. Additionally, *P*. *clavata* reaches maturity when colonies are from 3 years old (data from La Spezia population, [70]) to 7 years old (data from Medes Islands, Spain, [71]) and therefore only a few generations may have appeared after the mortality.

Our dispersal estimate (IBD regression slope of 0.037, R^2^ = 0.78) was similar to the one reported previously for populations from the whole Mediterranean, when IBD slope was 0.033 (R^2^ = 0.51) [29]. However, when comparing our results with a study that investigated a small spatial scale (IBD slope of 0.012, R^2^ = 0.85 [31]), the isolation by distance appears stronger in our study, implying a lower dispersal. Variability in dispersal estimates may arise form larval responses to environmental conditions, modifying planktonic larval duration (PLD), or from differential establishment success after dispersal. Indeed, PLD of up to 25 days, estimated from laboratory experiments [18], indicate that larvae can delay their metamorphosis when lacking the necessary settlement stimuli [65] and, therefore, may be capable of long distance dispersal. The demographic state of the local receiver populations may strongly condition the success of establishment of long distance migrants. In mature populations, the rare arrival of few migrants may pass unnoticed, lost among the high recruitment mortality bottleneck that is typical of most populations with overlapping generations. Therefore large-scale dispersal is rarely detected as effective gene flow. However, where mass mortality has occurred, the opportunity of expansion in freely available habitat magnifies the probabilities of success of long distance dispersers. Moreover, the study of [31] examined not only populations that experienced mortality, but also one population that was recently founded. These processes, which have been named density-barrier effects or prior colonization effects, are most strikingly demonstrated for species which have been able to rapidly expand for thousands of km along novel available habitat (e.g., in invasive expansions or postglacial recolonizations) whereas in the ancient native ranges they remain highly structured even across a few tens of km [72; 73]. Therefore, inferring probability of connectivity from genetic data alone may be misleading, where the demographic conditions of the populations may prevail over dispersal capabilities in determining the possibilities of population recovery by migration in a metapopulation.

The larval exchange between deep and shallow reefs, such as that inferred in Punta Mesco and Livorno, has significant importance for the species' conservation. Colonies dwelling below 25–30 m were not affected by mass mortality events, in contrast to shallow subpopulations [22; 25]. Our research supports the hypothesis of [27; 28] that deeper subpopulations may supply larvae to shallower sites. However, depth differences in the present study were relatively small, aimed at representing nearby areas above and below the thermocline warming effects. Further studies on larval dispersal, including deeper subpopulations, are needed to develop a more complete picture of larval exchange between different depths. Deeper reefs may have an even greater larval contribution for the shallower and other deep reefs than what we estimated here, especially after disturbances. In the STRUCTURE analysis, some of the migrants exchanged between Punta Mesco and La Spezia were grouped into a separate cluster when k = 4. They might have originated from a different population, possibly from a greater depth at Punta Mesco, where *P*. *clavata* occurs down to 60 m. The evidence for longer larval dispersal, between Punta Mesco and La Spezia, suggests that damaged reefs may benefit from external larval sources. Unexpectedly high numbers of recruits in the La Spezia population were observed shortly after the mass mortality event [24]. In the subsequent years recruits density was five times higher than in the pre mortality period [33]. Our findings indicate that not only increased reproductive output might be responsible for the recovery of the damaged population, but also larval migration.

## Conclusions

Our study failed to detect any genetic diversity loss in the *P*. *clavata* populations affected by mass mortality events. This may be due to the lack of test power and other confounding factors, including recent expansion or Wahlund effects. Our research confirmed low larval dispersal capability in the red gorgonian, since the maximum dispersal distance inferred from our data was between 20 and 60 km. However, this reduced ability for dispersal during the larval phase may still be ecologically significant for population replenishment and persistence, enabling migration between local populations. Population recovery after mortality events may be dependent on the possibility of propagule immigration from external sources, such as Protected Areas. Additionally, migration between reefs located at different depths implies that deeper refugia may provide larvae for shallow subpopulation recovery after climatically-induced mortality events affecting mostly shallow sites.

## Supporting Information

S1 Fig The plot of the LnP(D). (PDF)

S2 Fig The plot of Evanno deltaK. (PDF)

S1 Table Microsatellite data set. The allele sizes in 10 loci studied in 6 *P*. *clavata* populations in the Ligurian Sea.
(XLSX)
