# Supplementary figures and images for: Genetic Diversity and Local Connectivity in the Mediterranean Red Gorgonian Coral after Mass Mortality Events
Source: PLoS One. 2016 Mar 16;11(3):e0150590. doi: 10.1371/journal.pone.0150590 (PMC4794161; doi:10.1371/journal.pone.0150590)

L(K) (mean  $\pm$  SD)

Mean of est. Ln prob of data

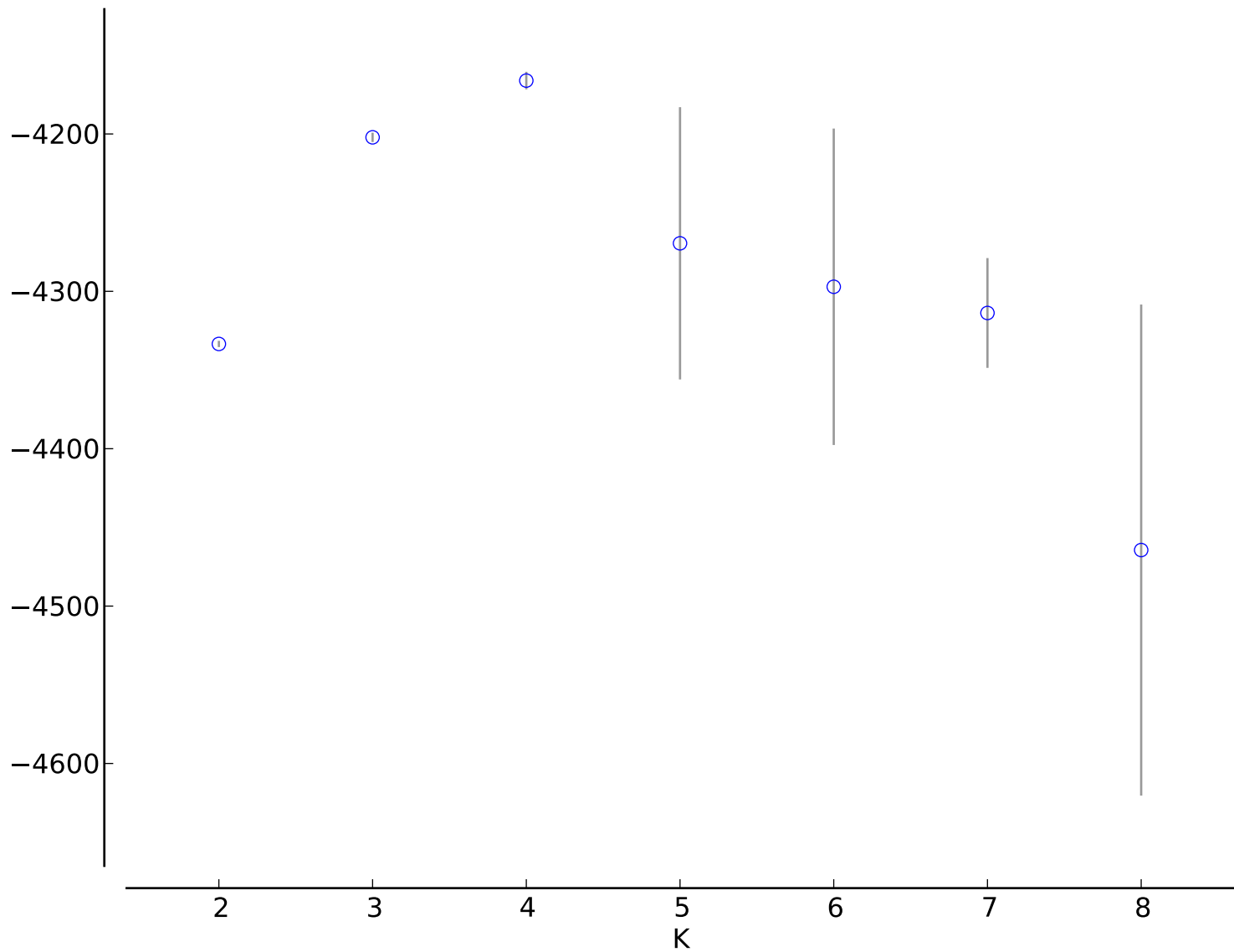

Supplement: S1 Fig — (PDF) [file pone.0150590.s001.pdf]

$$\text{DeltaK} = \text{mean}(|L''(K)|) / \text{sd}(L(K))$$

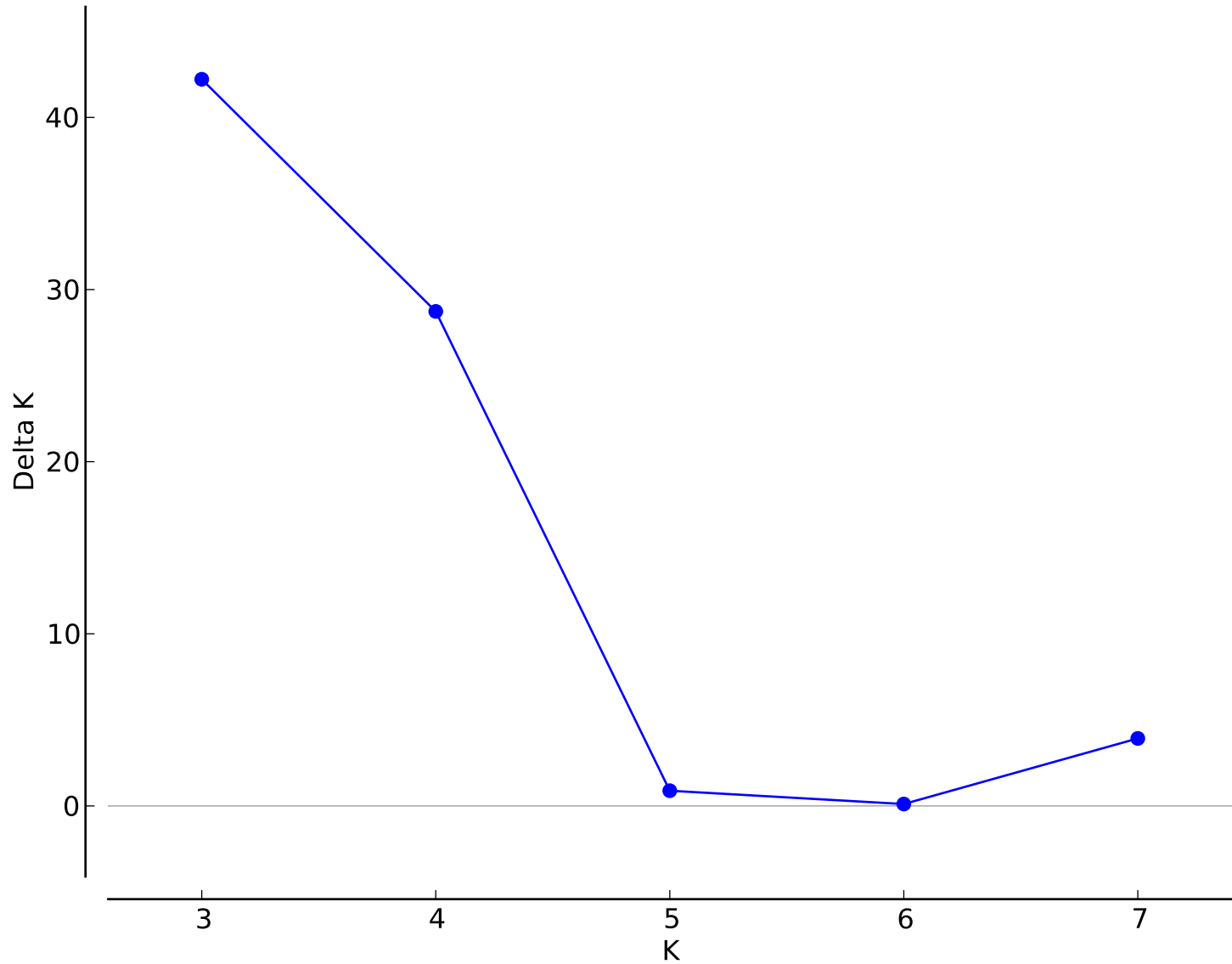

Supplement: S2 Fig — (PDF) [file pone.0150590.s002.pdf]
